# Preconditioning somatothermal stimulation on Qimen (LR14) reduces hepatic ischemia/reperfusion injury in rats

**DOI:** 10.1186/1472-6882-14-18

**Published:** 2014-01-13

**Authors:** Cheng-Chu Hsieh, Shu-Chen Hsieh, Jen-Hwey Chiu, Ying-Ling Wu

**Affiliations:** 1 Department and Institute of Veterinary Medicine, School of Veterinary Medicine, National Taiwan University, Taipei 106, Taiwan; 2 Biologics Division, Animal Health Research Institute, Council of Agriculture, Executive Yuan, New Taipei City 251, Taiwan; 3 Institute of Food Science and Technology, National Taiwan University, Taipei 106, Taiwan; 4 Division of General Surgery, Department of Surgery, Veterans General Hospital, Taipei 112, Taiwan; 5 Institute of Traditional Medicine, School of Medicine, National Yang-Ming University, Taipei 112, Taiwan

**Keywords:** Ischemia-reperfusion, Qimen (LR14), Superoxidase dismutase, Myeloperoxidase

## Abstract

**Background:**

In human beings or animals, ischemia/reperfusion (I/R) injury of the liver may occur in many clinical conditions, such as circulating shock, liver transplantation and surgery and several other pathological conditions. I/R injury has a complex pathophysiology resulting from a number of contributing factors. Therefore, it is difficult to achieve effective treatment or protection by individually targeting the mediators. This study aimed at studying the effects of local somatothermal stimulation preconditioning on the right Qimen (LR14) on hepatic I/R injury in rats.

**Methods:**

Eighteen male Sprague-Dawley rats were randomly divided into three groups. The rats were preconditioned with thermal tolerance study, which included one dose of local somatothermal stimulation (LSTS) on right Qimen (LR14) at an interval of 12 h, followed by hepatic ischemia for 60 min and then reperfusion for 60 min. Serum aspartate aminotransferase (AST) and alanine aminotransferase (ALT) have been used to assess the liver functions, and liver tissues were taken for the measurements such as malondialdehyde (MDA), glutathione (GSH), catalase (CAT), superoxidase dismutase (SOD), and myeloperoxidase (MPO).

**Results:**

The results show that the plasma ALT and AST activities were higher in the I/R group than in the control group. In addition, the plasma ALT and AST activities decreased in the groups that received LSTS. The hepatic SOD levels reduced significantly by I/R injury. Moreover, the hepatic MPO activity significantly increased by I/R injury while it decreased in the groups given LSTS.

**Conclusions:**

Our findings show that LSTS provides a protective effects on the liver from the I/R injury. Therefore, LSTS might offer an easy and inexpensive intervention for patients who have suffered from I/R of the liver especially in the process of hepatotomy and hepatic transplantation.

## Background

In human beings or animals, ischemia-reperfusion (I/R) injury of the liver may occur in many clinical conditions, such as circulating shock [1], disseminated intravascular coagulation [2], liver transplantation and surgery [3], cardiac failure and arrest, alcohol toxicity and several other pathological conditions. Different injury mechanisms contribute to the overall pathophysiology of the hepatic I/R injury. Because the liver is a highly oxygen-utilizing organ; therefore, the impairment of blood flow will rapidly cause hepatic hypoxia, which may progress to absolute anoxia especially in the pericentral regions of the liver lobe [4,5]. Therefore, it is very important to investigate the proper treatments for the I/R injury of the liver.

Oxygen-derived free radicals play a crucial role in I/R injury in many organs such as the heart and the liver [6,7]. Among the systemic consequences of reperfusion, lipid peroxidation is probably the most severe effect caused by free radicals, which results in structural and functional derangement and death of cells eventually [8,9]. Several lines of evidences have shown that HSP70 is a general anti-apoptotic protein, which protects cells from cytotoxicity induced by oxidative stress or chemotherapeutic agents [10].

Among the numerous complementary and alternative medicine therapies, acupuncture and moxibustion are ancient Chinese therapies that are most widely accepted as in the treatment of many diseases. There have been various studies on the mechanisms and efficacy of acupuncture and moxibustion. In addition, many researchers using the protocols of evidence-based medicine have proved that acupuncture is effective in treating chronic low back pain and digestive disorders [11], and also found moxibustion preconditioning protects the ischemic and anoxic brain tissue by increasing the activity of endogenous antioxidase [12]. Moreover, many hypotheses have been proposed to address the physiological mechanisms mediated by the action of acupuncture and moxibustion. In general, a large number of scientific studies have recently established strong evidence on the higher efficacy of acupuncture and moxibustion based on their clinical use over millenniums of years than that of the traditional treatment [13-15].

There are many modalities, including chemical and physical preconditioning, used to protect the organs from I/R injury. Massage, acupressure, chiropractic health care, exercise and hyperthermia *et al*. have been employed as treatment strategies for neuromuscular disorders or other diseases [16-19]. Recently, preconditioned local somatothermal stimulation (LSTS) has been proved to protect the heart and liver, increaseing the neuromuscular plasticity against I/R injury [20]. Therefore, LSTS is a maneuver similar to local heat therapy or the moxibustion technique in traditional Chinese medicine. Moreover, previous studies have demonstrated that the preconditioned LSTS on the right seventh intercostal nerve territory could increase hepatic HSP70 synthesis and protect the liver from I/R injury in rats [21]. Interestingly, the critical temperature evoking such visceral responses was around 41°C to 43°C, which was similar to those inducing HSP expression in a wide range of studies [21-24]. The induction of HSPs may afford protection from subsequent insults by decreasing leukocyte infiltration of postischemic tissues [25].

In our previous study, there are two possibilities to explain a somatovisceral reflex, namely, meridian theory and neuro-reflex theory. In meridian theory, Qimen (LR14) is an acupoint in the liver meridian. Accumulating evidence has demonstrated that there are convergent connections between somatic- and visceral neurons of the same or similar nerve territory during embryonic development. Besides, there is evidence that stimulation on an acupoint would regulate the corresponding organs through a complete loop of somatovisceral reflex, including receptors, afferent limbs, central system, efferent limbs and the target organ. Our previous work has demonstrated that the visceral response inhibited by blocking the afferent limb was blocked by local anesthesia [23]. Nonetheless, the possibility of the involvement of meridian theory can not be completely ruled out. In this study, we proved that the application of LSTS to the right Qimen (LR14) could induce the hepatic gene expression of HSP70, and this paper mainly focuses on whether LSTS passes through different pathway to protect the liver against subsequent I/R injury?

## Methods

### Animals

Eighteen male Sprague-Dawley rats each weighing 250–300 g, had been obtained from the animal center of the National Science Council, Taiwan, Republic of China. They were fed with standard diets and water *ad libitum* and treated under the regulations of the “Guide for the Care and Use of Laboratory Animals” (National Academy Press; 2011). The studies of these rats were approved by the ethics committee for animal study of National Yang-Ming University, Taipei, Taiwan, Republic of China.

### Local heat stress by local somatothermal stimulation

Application of local heat stress instead of using whole-body hyperthermia had been achieved by means of LSTS on skin areas with no direct contact with the skin surface. Conventional application of local heat therapy is supposed to evoke multiple sensory stimulation such as temperature, pressure, pain, and touch. In order to avoid causing any interference with the data interpretation by such multisensory stimulation, temperature was used as the sole stimulator in this study. The effect of LSTS done by using a heat generator (120 Watt. electric heating rod, Jise Co.) had been reported in our previous study regarding its relaxation effects on the sphincters of Oddi in cats, rabbits and the internal sphincters in rabbits [22,24]. In brief, LSTS had been achieved by the application of a heat generator supplying a single heat source on and above the skin areas when the skin temperature fluctuated above and below the critical point of 41°C. This was done by intermittently turning the switch of the heat generator on and off, namely, 4 min on and 5 min off for three courses [23]. In total, one dose of LSTS was completed in 27 min. A tinfoil paper with holes of 0.5 cm in diameter had also been used so that the heat generator could just focus on one point above the skin surface without insulation. Finally, we used a digital displayed thermometer to confirm that the critical heat point (41°C) was reached.

### Preconditioning by LSTS

**Figure 1:**
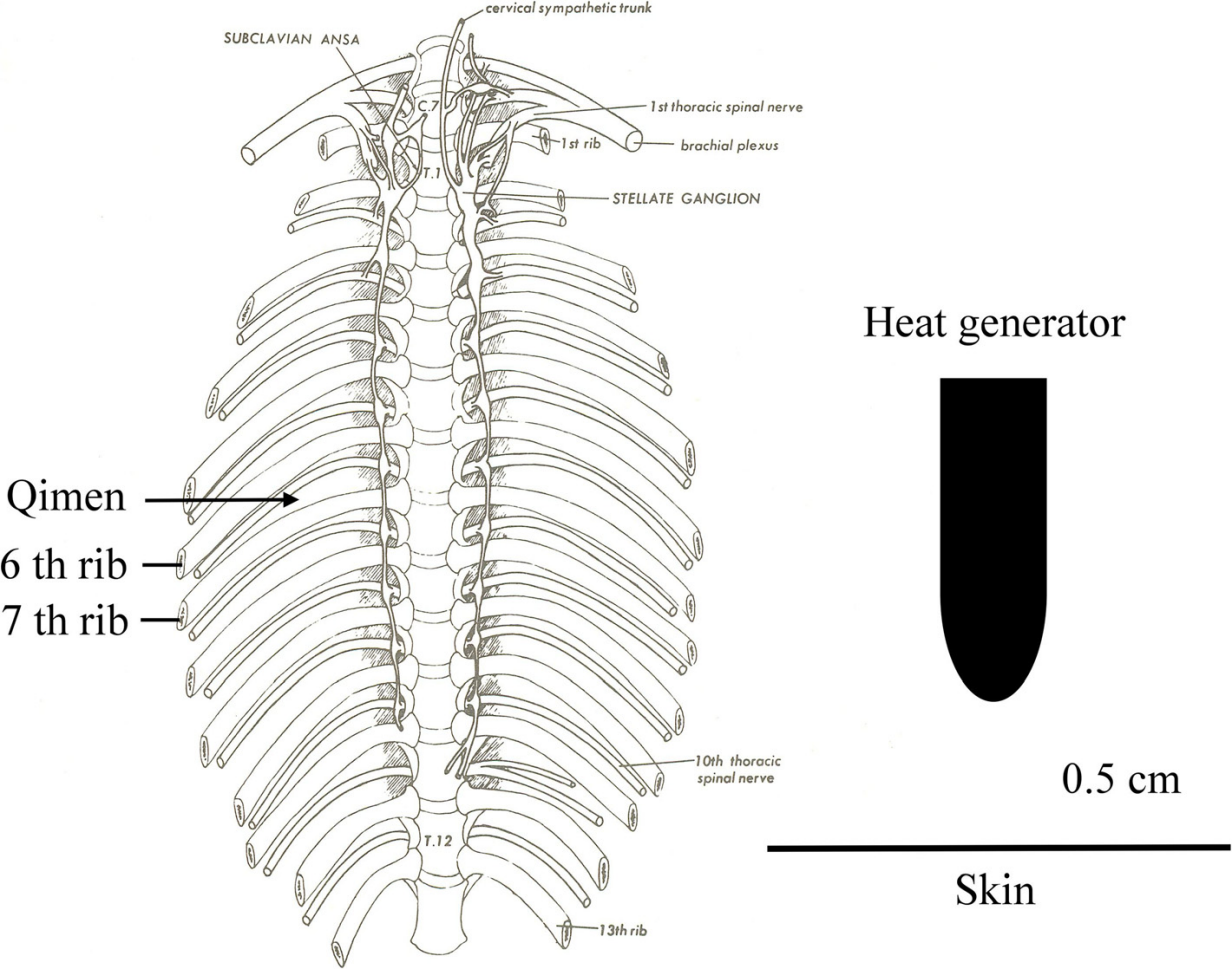
**Anatomical and acupoint position.** The heat generator was applied to 0.5 cm above the right Qimen (LR14), just at the junction of right midclavicular line and the sixth intercostal space.

To study the modulatory effect of preconditioned LSTS on the livers of I/R injury, the heat generator was applied to 0.5 cm above the right Qimen (LR14), which was just at the junction of the right mid-clavicular line and the sixth intercostal space (Figure 1). The rats were anesthetized with Ketamine (50 mg/kg, intraperitoneal injection (IP)), and the skin was shaved at the stimulus application point. The preconditioning LSTS was applied 12 h prior to the I/R liver injury.

### Animal model and parameters for the I/R injury of the liver

The model of rats with I/R injury was created as previously described [26,27]. In brief, male Sprague-Dawley rats were anesthetized with urethrane (1.25 g/kg, IP), and the trachea was cannulated for artificial respiration with a ventilator. Polyethylene (PE-50) catheters were cannulated into the femoral artery to monitor the blood pressure with a polygraph (Gould, RS 2400). The liver was exposed through an upper midline incision, and two pieces of fine silk were looped along the right and left branches of the portal vein, hepatic artery, and bile duct. The procedure whereby the silk was inserted into a snare with a piece of polyethylene (PE-90) which allowed the occlusion of the blood supply to either the median and left lobes (left branch). For the I/R injury study, ischemia of the median/left lobes were maintained for 60 min followed by reperfusion of the median/left lobes with immediate occlusion of the right lobe vasculature for another 60 min. One hour after the completion of the reperfusion procedure, the initial ischemic-reperfused median/left lobes were resected and tested for myeloperoxidase (MPO), malondialdehyde (MDA), glutathione (GSH), catalase (CAT), superoxidase dismutase (SOD) measurement. Blood samples for ALT/AST measurement were collected immediately after the femoral catheterization and the completion of the reperfusion procedure.

### Experimental groups

Eighteen male Sprague-Dawley rats were randomly divided into three groups, which were the: control, I/R and I/R + LSTS groups, where each group had six rats (n = 6). In the control group, none of the rats were treated with LSTS, nor subjected to the I/R liver injury. In the I/R group, the rats were subjected to 60 min of hepatic ischemia followed by 60 min of reperfusion period. In the I/R + LSTS group, the rats were preconditioned for thermal tolerance study, which included one dose of LSTS on the right Qimen (LR14) at an interval of 12 h then hepatic ischemia for 60 min followed by reperfusion for 60 min.

### Biochemical analysis

#### *Hepatic SOD assay*

The hepatic SOD activity was determined by using a commercialized chemical SOD assay kit (Cayman Chemical Co.). The kit utilizes a tetrazolium salt for the detection of superoxide radicals generated by xanthine oxidase and hypoxanthine. The collected 1 gm of liver tissues were homogenized in 5 ml of cold buffer (20 mM HEPES buffer, pH 7.2, containing 1 mM EDTA, 210 mM mannitol, and 70 mM sucrose) and centrifuged at 1,500 × *g* for 5 min at 4°C. The reaction was initiated by adding xanthine oxidase and incubating at room temperature for 20 min, and then, the absorbance of each sample was read at 450 nm.

#### *Hepatic CAT assay*

The CAT activity was determined by using a commercialized chemical CAT assay kit (Cayman Chemical Co.). The kit utilizes the peroxidatic function of CAT for the measurement of enzyme activity. The collected 1 gm of liver tissues were homogenized in 5 ml of cold buffer (50 mM potassium phosphate, pH 7.0, containing 1 mM EDTA) and centrifuged at 10,000 × *g* for 15 min at 4°C. The samples were then mixed sequentially with hydrogen peroxide; potassium hydroxide, Purpald**®**, and potassium periodate and were read at 540 nm.

#### *Hepatic GSH assay*

The hepatic levels of GSH were determined by using a commercialized GSH assay kit (Cayman Chemical Co., Ann Arbor, MI, USA). Cayman’s GSH assay kit utilizes a carefully optimized enzymatic recycling method which uses glutathione reductase for the quantification of GSH. The collected 1 g of liver tissues were first homogenized in 5 ml of cold buffer (i.e., 50 mM MES or phosphate, pH 6–7, containing 1 mM EDTA) and centrifuged at 10,000 × *g* for 15 min at 4°C followed by metaphosphoric acid deproteinization. After adding triethanolamine solution and Assay Cocktail [a mixture of MES buffer (11.25 ml), reconstituted cofactor mixture (0.45 ml), reconstituted enzyme mixture (2.1 ml), water (2.3 ml), and reconstituted DTNB (0.45 ml)], total GSH in each of the deproteinated samples was measured at 405 nm.

#### *Hepatic MPO assay*

MPO activity was measured from liver tissues using a procedure similar to that documented by Hillegas *et al*. [28]. The collected 1 g of liver tissue samples were homogenized in 15 ml of potassium phosphate buffer (PB, 50 mM, pH 6.0) and centrifuged at 41,400 × g for 10 min. The pellets were then suspended in 50 mM PB containing 0.5% hexadecyl trimethyl ammonium bromide (HETAB). After three freezing and thawing cycles with sonication between cycles, the samples were next centrifuged at 41.400 × *g* for 10 min. Each aliquot (0.3 ml) was then added to 2.3 ml of a reaction mixture containing 50 mM PB, 0.19 mg/ml o-dianisidine, and 20 mM H_2_O_2_ solution. One unit of enzyme activity had been defined as the amount of the MPO that caused a change in the absorbance measured at 460 nm for 2 min. MPO activity had been expressed as U/g tissue.

#### *Hepatic lipid peroxidation assay*

The collected 0.5 g of liver tissue samples were homogenized with 0.01 M sodium phosphate buffer (pH 7.0) 4.5 ml. After the addition of 1% phosphoric acid 1.5 ml and 0.6% TBA solution 0.5 ml, the solution were mixed evenly, and then heated at 95°C for 1 h. Phosphorylation of lipid peroxides to be hydrolyzed by boiling, and hydrolysis products of the MDA with TBA (2-Thiobarbituric acid) to produce MDA (TBA)_2_. Then cool to room temperature, add 2 ml of butanol (C_4_H_10_O), and centrifuged at 2,000 × g for 15 min, take butanol layer for ELISA reader at 532 nm absorbance was measured, and TEP (1,1,3,3-tetraethoxypropane) as the standard value.

#### *Histological analysis*

The rats were sacrificed by decapitation, and small pieces of liver tissues were placed in 10% (vol/vol) formaline solution and processed routinely by embedding in paraffin. Tissue sections (4–5 μm) were stained with Hematoxylin & Eosin (H & E) and examined under a light microscope.

#### *Data analysis*

The data in each experimental group were analyzed and expressed as means ± standard error of the mean (SEM). Concentrations of MPO, MDA, GSH, CAT, SOD, ALT, and AST in different groups were determined by using the Wilcoxon rank-sum test to compare the differences between two experimental groups. A *P* value less than 0.05 indicates statistical significance. Error bars indicates ± SEM in the figure.

## Results

### Hepatic I/R induction and effects of LSTS on Qimen (LR14)

During the experimental procedure, blood pressure was continuously monitored using a polygraph. The blood pressure of all experimental rats remained stable, and none of them died during I/R induction. To evaluate the level of liver damage after the reperfusion procedure, blood samples from different groups were collected immediately via the femoral catheters after completing the reperfusion procedure. As shown in Table 1, the ALT and AST levels increased significantly in the I/R group as compared with the control group, while treatment with LSTS ameliorated the increases in ALT and AST. These results indicated that the liver damage had been attenuated.

**Table 1:** Plasma ALT and AST activities

**Experimental groups**	**ALT (U/L)**	**AST (U/L)**
Control	74 ± 11.2	109 ± 7.2
I/R	843 ± 131^*^	1457 ± 263^*^
I/R + LSTS	505 ± 75^*,+^	549 ± 137^*,+^

^*^P < 0.05: Compared with the control group.

^*+*^*P* < 0.05: Compared with the I/R group.

The results showed that the plasma ALT and AST activities were higher in the I/R group than in the control group.

### The antioxidant activity of LSTS on Qimen (LR14) in the liver

**Figure 2:**
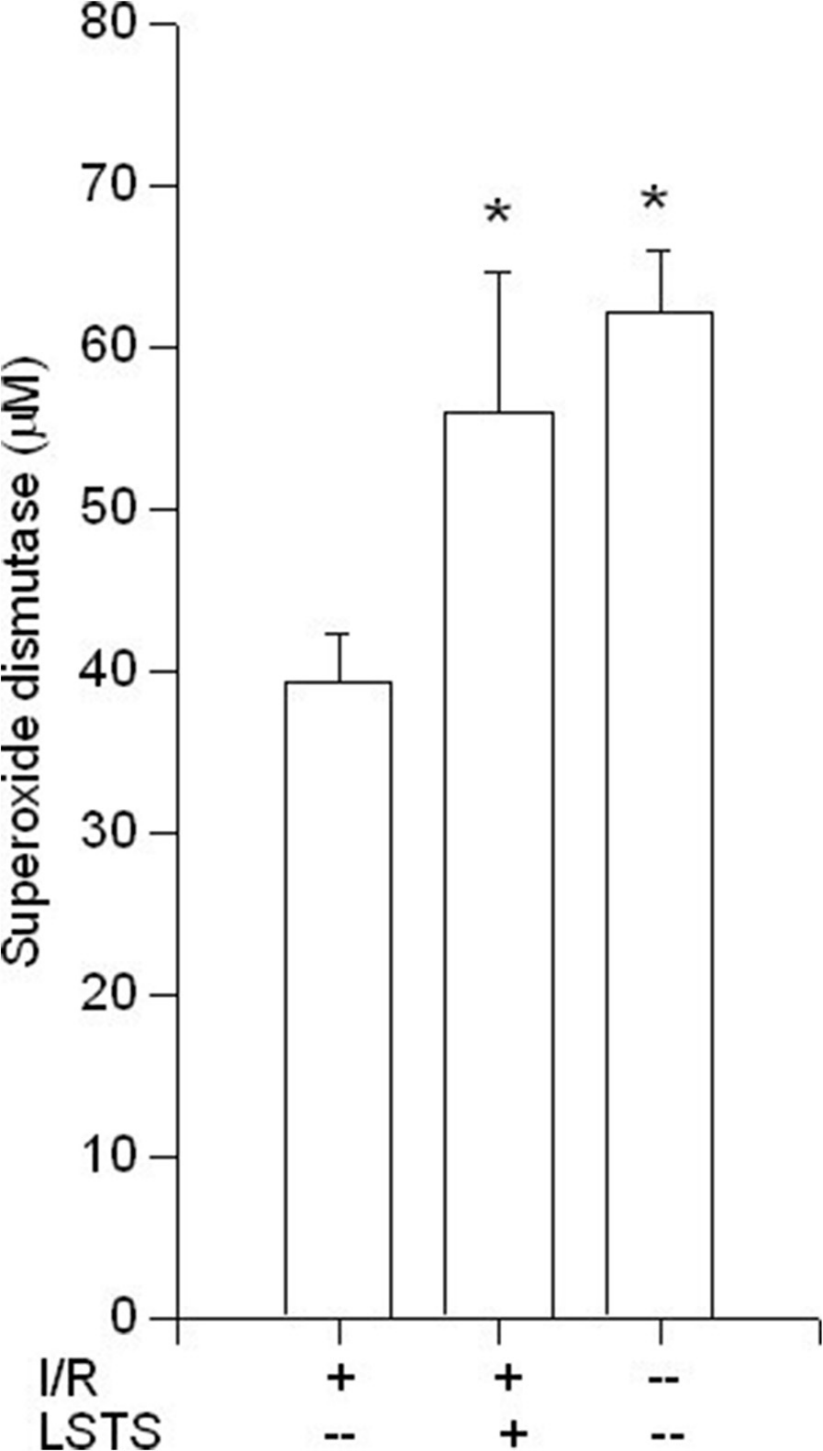
**Hepatic SOD results.** Effects of ischemia/reperfusion and its pre-treatment with LSTS on SOD levels (SOD) of collected liver tissue. ^*^P < 0.05: Compared to the I/R group.

**Figure 3:**
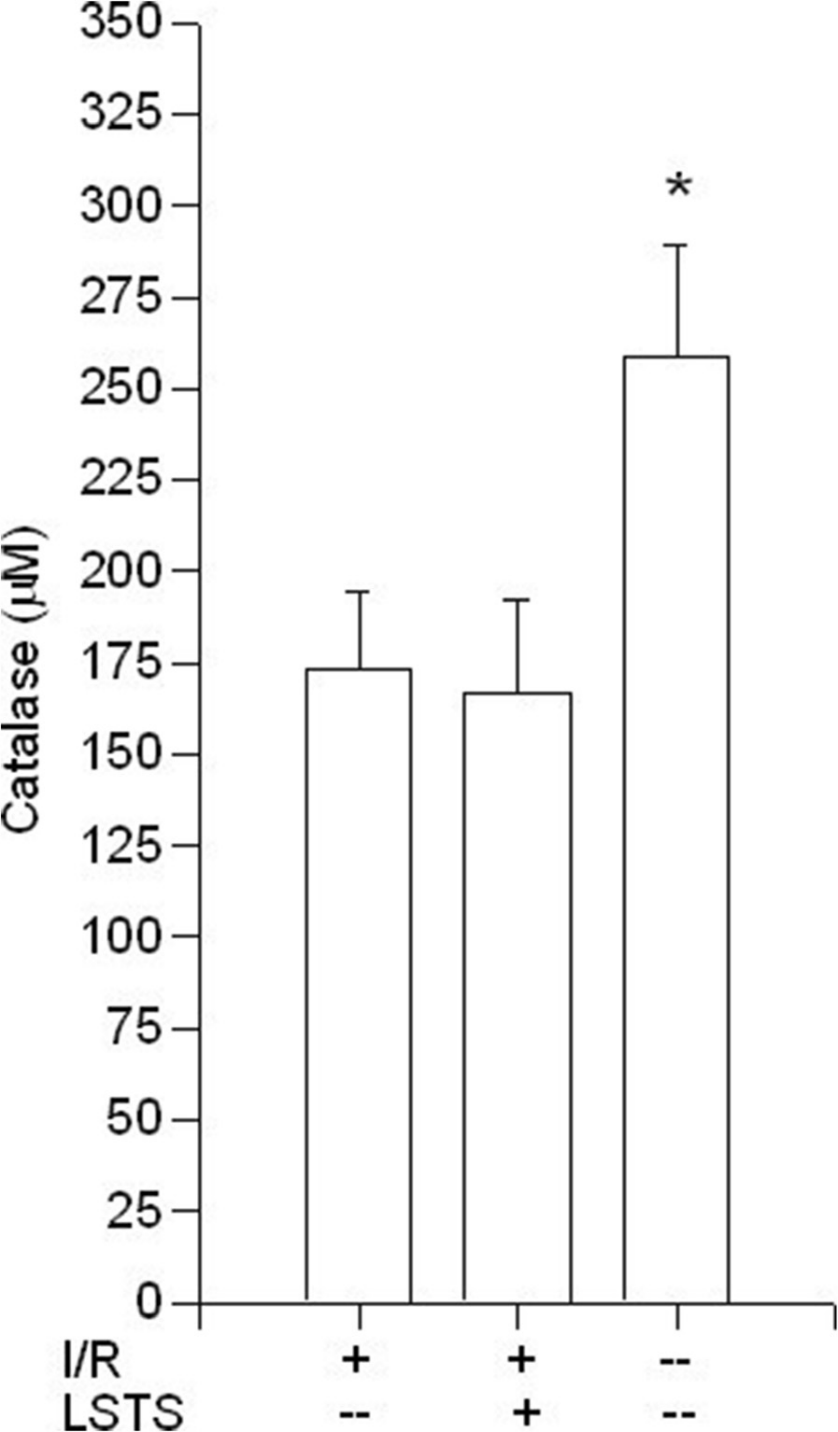
**Hepatic CAT results.** Effects of ischemia/reperfusion and its pre-treatment with LSTS on CAT levels of collected liver tissue. ^*^P < 0.05: Compared to the I/R group.

**Figure 4:**
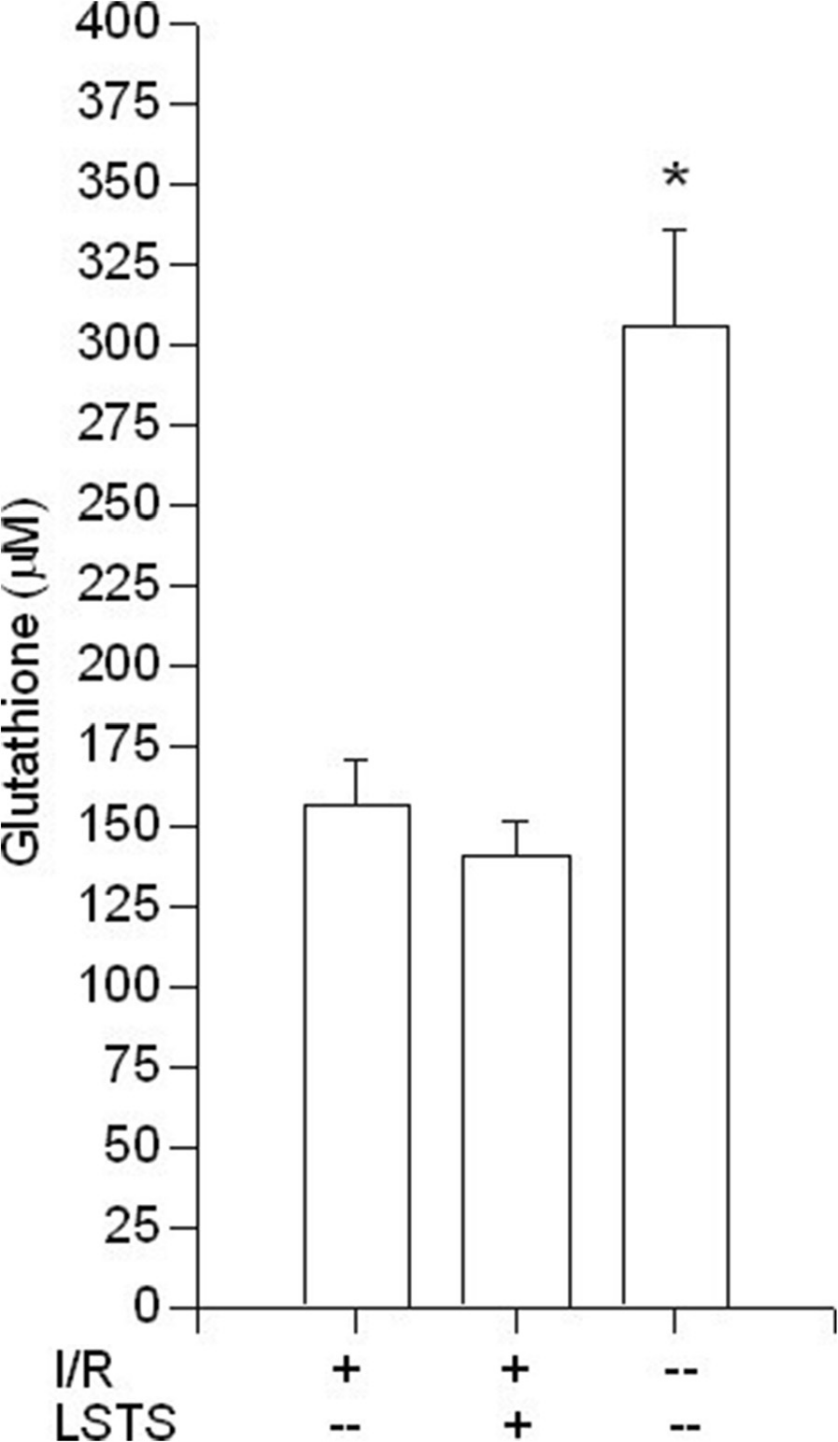
**Hepatic GSH results.** Effects of ischemia/reperfusion and its pre-treatment with LSTS on GSH levels of collected liver tissue. ^*^P < 0.05: Compared to the I/R group.

**Figure 5:**
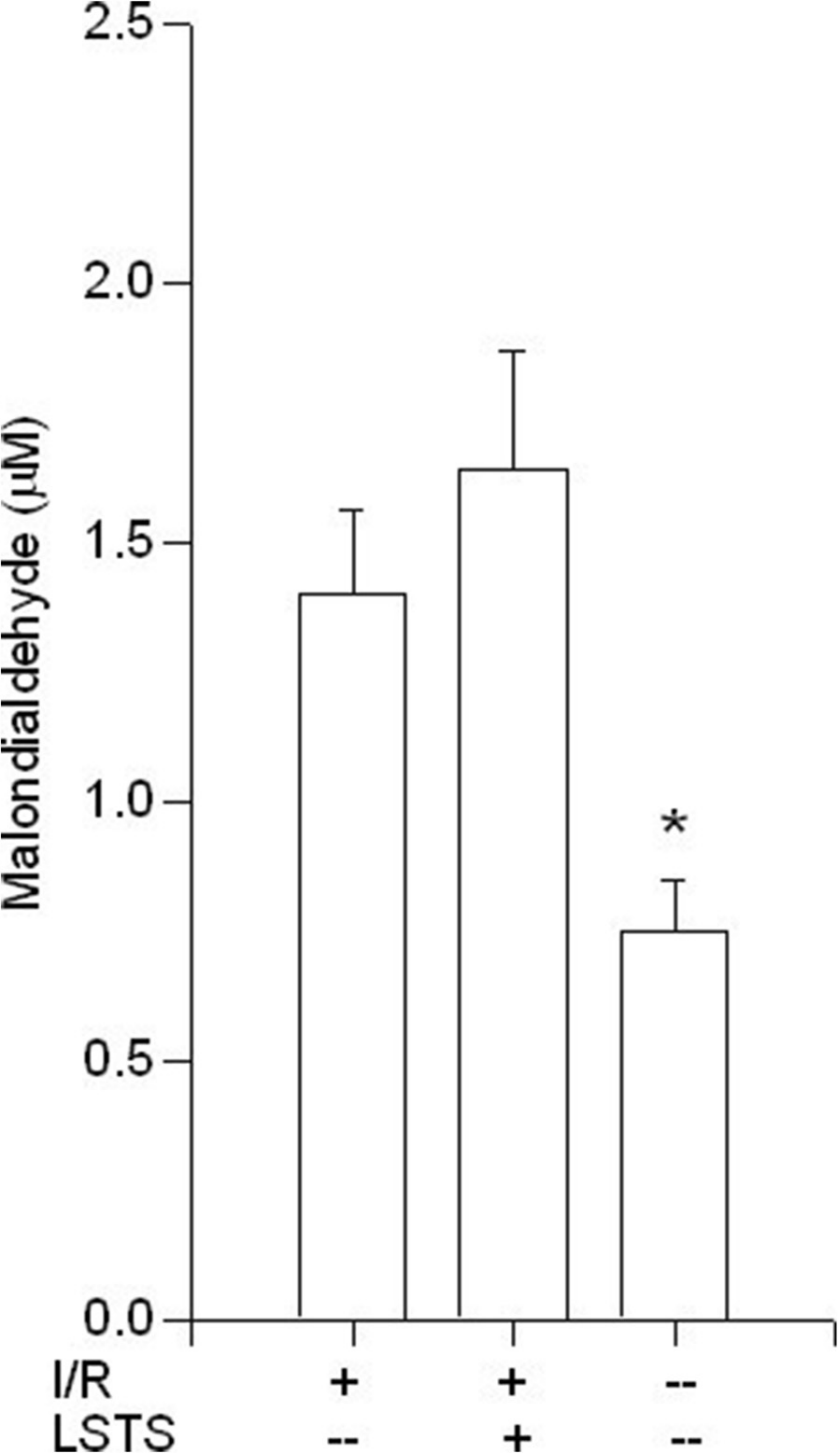
**Hepatic MDA results.** Effects of ischemia/reperfusion and its pre-treatment with LSTS on MDA levels of collected liver tissue. ^*^P < 0.05: Compared to the I/R group.

Because antioxidant enzymes are important for maintaining an optimal chemical reducing environment to prevent liver from reactive oxygen species (ROS) damage; thus, we analyzed the activities of several hepatic enzymes. As shown in Figure 2, SOD levels were significantly decreased after I/R injury when compared with the control group. Compared with the I/R group, LSTS treatments increased the SOD levels (*P* < 0.05). The level of hepatic CAT in the liver tissue also decreased after I/R induction in comparison with control group. Compared with I/R group, LSTS treatments revealed comparable amount of CAT (Figure 3). We also measured the level of GSH, which participates in many metabolic processes that protect cells against the actions of free radicals. As shown in Figure 4, the hepatic GSH levels was significantly lower in I/R group than in the control group, whereas the intervention of LSTS in I/R treated mice showed no effects on the level of GSH when compared with I/R group. The mean liver MDA level, an indicator of lipid peroxidation, was also estimated. The MDA value was significantly higher in the I/R group than in the control group. Compared with the I/R group, the mean MDA level was no significant difference in the LSTS group (Figure 5).

### The anti-inflammatory effect of LSTS on Qimen (LR14) in the liver

**Figure 6:**
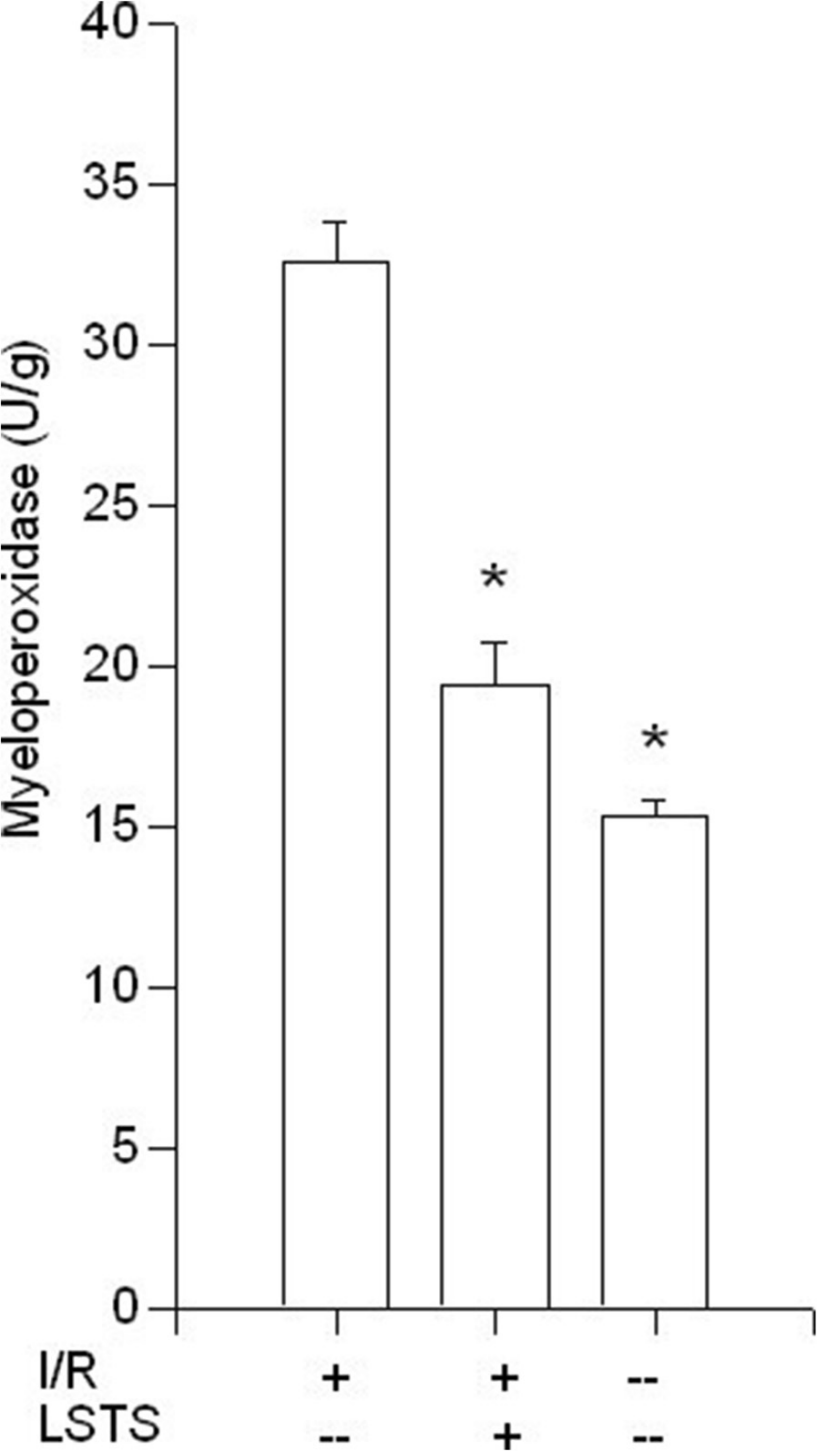
**Hepatic MPO results.** Effects of ischemia/reperfusion and its pre-treatment with LSTS on MPO levels of collected liver tissue. ^*^P < 0.05: Compared to the I/R group.

Because the inflammatory response after I/R plays a critical role in cell damage, we examined the hepatic MPO levels. This enzyme is reflective of tissue infiltration with neutrophils. As shown in Figure 6, the mean hepatic MPO level in the I/R group was significantly increased in comparison to the control group. The MPO level in the I/R group that received LSTS treatment was lower compared with the I/R group (*P* < 0.05).

### Histological results

**Figure 7:**
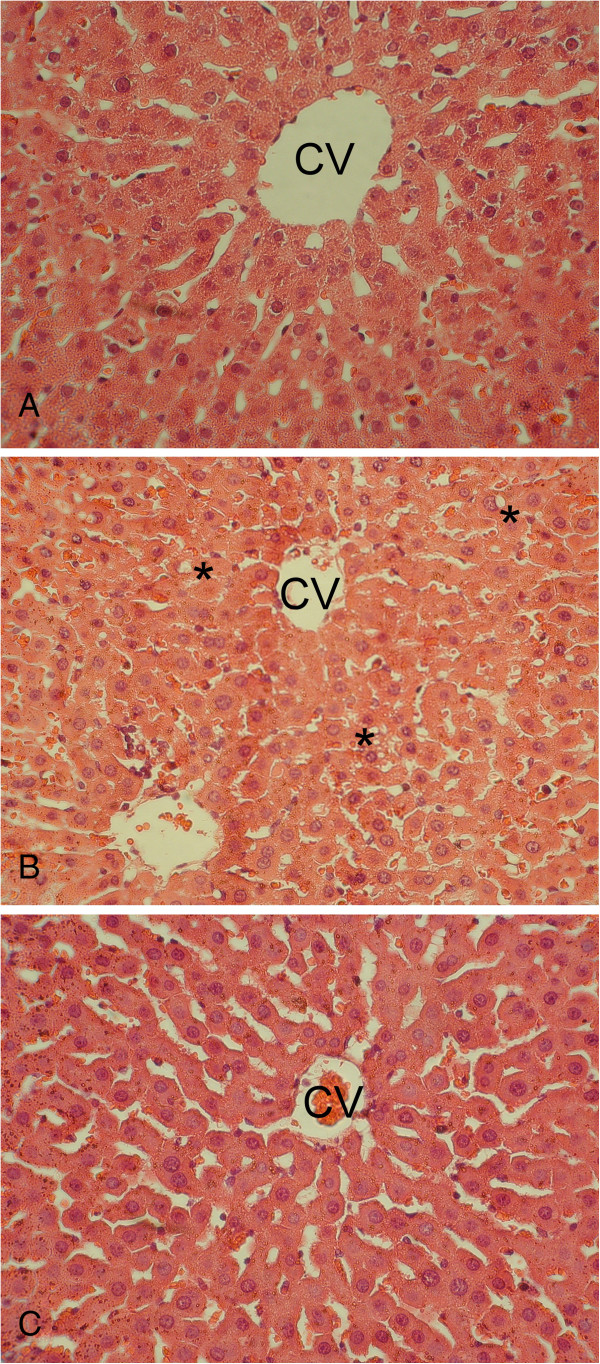
**Histological results. A**. In the control group, the normal liver parenchyme occurred showed regular morphology of both hepatocytes and sinusoids around the central vein (CV). **B**. In the I/R group, hepatocytes (*) were prominently swollen with marked vacuolization. Congestion was noticed in enlarged sinusoids. The liver parenchyme took place displayed irregular morphology of both hepatocytes and sinusoids around the central vein. **C**. In the I/R + LSTS group, hepatocytes and sinusoids represented normal morphology reflecting a well preserved liver parenchyma (400X).

In the control group, the normal liver parenchyma showed regular morphology of both hepatocytes and sinusoids around the central vein. In the I/R group, the hepatocytes were prominently swollen with marked vacuolization. Congestions were noticed in enlarged sinusoids. The liver parenchyma were accompanied with irregular morphology of both hepatocytes and sinusoids around the central vein. In the I/R + LSTS group, the hepatocytes and sinusoids displayed normal morphology reflecting well preserved liver parenchyma (Figure 7).

## Discussion

LSTS has been reported to up-regulate the gene expression of HSP70 in livers and preconditioning with LSTS on the right seventh intercostal nerve territory can protect rat livers from I/R injury. In addition, hepatic HSP70 gene expression has been demonstrated to be regulated by local heat stress over a designated skin area through a somatovisceral reflex and not by whole-body hyperthermia [21,23].

With regard to the animal model used in this study, it is well-known that the right lobe vasculature occluded with the reperfusion of median/left lobes has been suggested to be a good model for studies on hepatic I/R injury [21,26,27]. In the studies on hepatic I/R injury, determinating the time for each I/R period is important. Ischemia for too-short (20 min) or too-long (90 min) time might result in little or irreversible structural and functional changes, respectively. Sinusoidal perfusion failure was found to be aggravated when the ischemic time period was prolonged to 60 min [29]. Using 60 min of median/left lobar ischemia followed by 60 min of reperfusion as a model, our results showed distinct functional alterations (Table 1) and this provided a reproducible system for studying the protective effect of LSTS on hepatocytes from I/R injury.

The I/R injury of livers can occurred in many clinical scenarios including transplantation, trauma and hepatectomy. The main pathophysiological events during this injury comprise depletion of ATP, Kupffer cell activation with subsequent formation of ROS, formation of proinflammatory mediators, and recruitment and activation of macrophages, neutrophils, and lymphocytes. Depending on the severity of the I/R injury, the cell damage can lead to necrotic or apoptotic liver cell death, which can subsequently result in organ dysfunction [30-32]. Oxygen-derived free radicals play an important role in I/R injury in many organs like the heart and liver. The aim of these treatments is to restore the blood supply to the ischemic liver. Unfortunately, restoration of the blood flow to ischemic tissues can lead to further damages. This paradox leads to uncertain effectiveness of these strategies. The I/R injury is a complex pathophysiology with a number of contributing factors; therefore, it is difficult to obtain effective treatments or protections by targeting individual mediators or mechanisms alone. Preconditioned fasting, ischemia, pharmaceutical molecules, hyperosmolar solutions, and LSTS [23,33-36] have been studied and appear to have the ability to increase the resistance of cells to ischemia and reperfusion events.

In this study, we clearly demonstrated that LSTS on the right Qimen (LR14) up-regulated the levels of SOD and down-regulated the level of MPO in the liver. SOD catalyses the dismutation of the superoxide anion (O_2_·) into H_2_O_2_, which could be transformed into H_2_O and O_2_ by CAT. In this study, we found that I/R impaired SOD activity as indicated by the markedly lowered activity when compared with the control group. But in the LSTS pretreated group, the decrease of SOD activity was significantly counteracted. HSP70 acts as a molecular chaperone, playing an essential role in mediating protein folding, assembly, transport, and degradation, helping to prevent protein denaturation and aggregation, and assisting in the refolding or removal of damaged proteins [37,38]. It has been shown that the SOD activity and its content decrease after the I/R injury presumably via inactivation of mature and active SOD within mitochondria [39]. It is, however, unlikely that HSP70 could prevent I/R injury induced denaturation or inactivation of mature SOD within mitochondria, because HSP70 localize in different compartments from mature and active SOD. Specifically, HSP70 distributes in cytoplasm and nucleus in both normal and stressed conditions [38,40], while the mature and active SOD exists and acts in mitochondria [41]. It has been clarified that HSP70 plays an essential role in keeping newly synthesized mitochondrial proteins (precursor proteins) in their correct unfolding conformation within the cytoplasm and transporting them into mitochondria [30,31]. Therefore, one should speculate that cytoplasmic HSP70 overexpression could enhance translocation of precursor SOD into mitochondria, which then results in supplementing the pool of mature SOD within these organelles. We have demonstrated that the liver HSP70 overexpression results in improved liver tolerance to I/R injury and is associated with enhanced preservation of the SOD activity.

MPO is an enzyme stored in azurophilic granules of polymorphonuclear neutrophils and macrophages, and released into extracellular fluid in the setting of inflammatory process. MPO is involved in acute and chronic inflammatory diseases. The significant increase in the MPO activity in the liver tissue after hepatic I/R in the present study is consistent with another study [42]. The induction of HSPs may afford protection from subsequent insults by decreasing leukocyte infiltration of post-ischemic tissues [25]. Javadpour *et al.* found fewer pulmonary neutrophils and less MPO activity after aortic occlusion in rats with prior induction of HSPs via hyperthermia [42] or pharmacologic means [43]. They also showed that hyperthermia prevented decrease in leukocyte rolling velocity induced by mesenteric I/R [44]. Prior hyperthermia also decreases intestinal neutrophil infiltration and mucosal injury after intestinal ischemia [45]. HSP70 may regulate MPO activates to mitigate the I/R injury. In additional, MPO can also serve as an indicator of inflammation and the generation of ROS, because an increase in MPO activity reflects tissue neutrophil infiltration. Neutrophils are a potential source of oxygen free radicals [46] and are considered to be the major effector cells involved in tissue damage that occurs in several inflammatory diseases [47,48]. Our results show that LSTS on Qimen (LR14) may decrease MPO activates to reduce hepatic I/R injury.

## Conclusions

Preconditioned LSTS on the right Qimen (LR14) have a beneficial effect on protecting the rat liver against I/R injury. This pre-treatment is more advantageous when compared with the pretreatment by means of whole-body hyperthermia. LSTS is an easily applicable alternative and will bring new perspectives to the clinical prevention of ischemic liver disease or liver transplantation.

## Abbreviations

I/R: Ischemia/reperfusion; LSTS: Local somatothermal stimulation; AST: Aspartate aminotransferase; ALT: Alanine aminotransferase; IP: Intraperitoneal injection; MDA: Malondialdehyde; GSH: Glutathione; CAT: Catalase; SOD: Superoxidase dismutase; MPO: Myeloperoxidase; ROS: Reactive oxygen species; H & E: Hematoxylin & Eosin.

## Competing interests

All authors declare that there are no financial competing interests.

## Authors’ contributions

Each of these authors has contributed equally to this article. CC carried out the I/R injury mechanism studies, acquisition of data, or analysis and interpretation of data, and drafted the manuscript. JH participated in the design of the study, performed the statistical analysis and helped to proofread the manuscript. SC and YL participated in its design and coordination and helped to draft the manuscript. All authors read and approved the final manuscript.

## Pre-publication history

The pre-publication history for this paper can be accessed here:

http://www.biomedcentral.com/1472-6882/14/18/prepub

## References

[B1] de la Monte SM Arcidi JM Moore GW Hutchins GM Midzonal necrosis as a pattern of hepatocellular injury after shock Gastroenterology 1984 86 4 627 631 6698364

[B2] Yoshikawa T Murakami M Yoshida N Seto O Kondo M Effects of superoxide dismutase and catalase on disseminated intravascular coagulation in rats Thromb Haemost 1983 50 4 869 872 6665768

[B3] Arthur MJ Reactive oxygen intermediates and liver injury J Hepatol 1988 6 1 125 131 10.1016/S0168-8278(88)80472-0 3279103

[B4] Jungermann K Kietzmann T Oxygen: modulator of metabolic zonation and disease of the liver Hepatology 2000 31 2 255 260 10.1002/hep.510310201 10655244

[B5] Lemasters JJ Ji S Thurman RG Centrilobular injury following hypoxia in isolated, perfused rat liver Science 1981 213 4508 661 663 10.1126/science.7256265 7256265

[B6] Gonzalez-Flecha B Cutrin JC Boveris A Time course and mechanism of oxidative stress and tissue damage in rat liver subjected to in vivo ischemia-reperfusion J Clin Invest 1993 91 2 456 464 10.1172/JCI116223 8432855 PMC287955

[B7] Halliwell B Reactive oxygen species in living systems: source, biochemistry, and role in human disease Am J Med 1991 91 3C 14S 22S 1928205 10.1016/0002-9343(91)90279-7

[B8] McCord JM Oxygen-derived free radicals in postischemic tissue injury N Engl J Med 1985 312 3 159 163 10.1056/NEJM198501173120305 2981404

[B9] Vollmar B Glasz J Leiderer R Post S Menger MD Hepatic microcirculatory perfusion failure is a determinant of liver dysfunction in warm ischemia-reperfusion Am J Pathol 1994 145 6 1421 1431 7992845 PMC1887483

[B10] Jaattela M Wissing D Kokholm K Kallunki T Egeblad M Hsp70 exerts its anti-apoptotic function downstream of caspase-3-like proteases EMBO J 1998 17 21 6124 6134 10.1093/emboj/17.21.6124 9799222 PMC1170939

[B11] Inoue M Kitakoji H Yano T Ishizaki N Itoi M Katsumi Y Acupuncture treatment for low back pain and lower limb symptoms-the relation between acupuncture or electroacupuncture stimulation and sciatic nerve blood flow Evid Based Complement Alternat Med 2008 5 2 133 143 10.1093/ecam/nem050 18604251 PMC2396470

[B12] Hua JS Li LP Zhu XM Effects of moxibustion pretreating on SOD and MDA in the rat of global brain ischemia J Tradit Chin Med 2008 28 4 289 292 10.1016/S0254-6272(09)60014-5 19226902

[B13] Pang Y Wu LB Liu DH Acupuncture therapy for apoplectic aphasia: a systematic review Zhongguo Zhen Jiu 2010 30 7 612 616 20862949

[B14] Berman BM Langevin HM Witt CM Dubner R Acupuncture for chronic low back pain. N Engl J Med 2010 363 5 454 461 10.1056/NEJMct0806114 20818865

[B15] Lee MS Choi TY Kang JW Lee BJ Ernst E Moxibustion for treating pain: a systematic review Am J Chin Med 2010 38 5 829 838 10.1142/S0192415X10008275 20821815

[B16] Beyerman KL Palmerino MB Zohn LE Kane GM Foster KA Efficacy of treating low back pain and dysfunction secondary to osteoarthritis: chiropractic care compared with moist heat alone J Manipulative Physiol Ther 2006 29 2 107 114 10.1016/j.jmpt.2005.10.005 16461169

[B17] Yeh CC Ko SC Huh BK Kuo CP Wu CT Cherng CH Wong CS Shoulder tip pain after laparoscopic surgery analgesia by collateral meridian acupressure (shiatsu) therapy: a report of 2 cases J Manipulative Physiol Ther 2008 31 6 484 488 10.1016/j.jmpt.2008.06.005 18722205

[B18] Arroyo-Morales M Olea N Martinez M Moreno-Lorenzo C Diaz-Rodriguez L Hidalgo-Lozano A Effects of myofascial release after high-intensity exercise: a randomized clinical trial J Manipulative Physiol Ther 2008 31 3 217 223 10.1016/j.jmpt.2008.02.009 18394499

[B19] Pfefer MT Cooper SR Uhl NL Chiropractic management of tendinopathy: a literature synthesis J Manipulative Physiol Ther 2009 32 1 41 52 10.1016/j.jmpt.2008.09.014 19121463

[B20] Pan PJ Chan RC Yang AH Chou CL Cheng YF Chiu JH Protective effects of preconditioned local somatothermal stimulation on neuromuscular plasticity against ischemia–reperfusion injury in rats J Orthop Res 2008 26 12 1670 1674 10.1002/jor.20693 18634018

[B21] Lin YH Chiu JH Tung HH Tsou MT Lui WY Wu CW Preconditioning somatothermal stimulation on right seventh intercostal nerve territory increases hepatic heat shock protein 70 and protects the liver from ischemia-reperfusion injury in rats J Surg Res 2001 99 2 328 334 10.1006/jsre.2001.6177 11469906

[B22] Chiu JH Lui WY Chen YL Hong CY Local somatothermal stimulation inhibits the motility of sphincter of Oddi in cats, rabbits and humans through nitrergic neural release of nitric oxide Life Sci 1998 63 6 413 428 10.1016/S0024-3205(98)00291-4 9718066

[B23] Chiu JH Tsou MT Tung HH Tai CH Tsai SK Chih CL Lin JG Wu CW Preconditioned somatothermal stimulation on median nerve territory increases myocardial heat shock protein 70 and protects rat hearts against ischemia-reperfusion injury J Thorac Cardiovasc Surg 2003 125 3 678 685 10.1067/mtc.2003.29 12658212

[B24] Jiang JK Chiu JH Lin JK Local somatothermal stimulation inhibits motility of the internal anal sphincter through nitrergic neural release of nitric oxide Dis Colon Rectum 2000 43 3 381 388 10.1007/BF02258306 10733121

[B25] Hiratsuka M Yano M Mora BN Nagahiro I Cooper JD Patterson GA Heat shock pretreatment protects pulmonary isografts from subsequent ischemia-reperfusion injury J Heart Lung Transplant 1998 17 12 1238 1246 9883766

[B26] Canada AT Stein K Martel D Watkins WD Biochemical appraisal of models for hepatic ischemia-reperfusion injury Circ Shock 1992 36 3 163 168 1611700

[B27] Chiu JH Ho CT Wei YH Lui WY Hong CY In vitro and in vivo protective effect of honokiol on rat liver from peroxidative injury Life Sci 1997 61 19 1961 1971 10.1016/S0024-3205(97)00836-9 9364201

[B28] Hillegass LM Griswold DE Brickson B Albrightson-Winslow C Assessment of myeloperoxidase activity in whole rat kidney J Pharmacol Methods 1990 24 4 285 295 10.1016/0160-5402(90)90013-B 1963456

[B29] Koo A Komatsu H Tao G Inoue M Guth PH Kaplowitz N Contribution of no-reflow phenomenon to hepatic injury after ischemia-reperfusion: evidence for a role for superoxide anion Hepatology 1992 15 3 507 514 10.1002/hep.1840150325 1312056

[B30] Deshaies RJ Koch BD Werner-Washburne M Craig EA Schekman R A subfamily of stress proteins facilitates translocation of secretory and mitochondrial precursor polypeptides Nature 1988 332 6167 800 805 10.1038/332800a0 3282178

[B31] Wienhues U Neupert W Protein translocation across mitochondrial membranes Bioessays 1992 14 1 17 23 10.1002/bies.950140105 1532121

[B32] Jaeschke H Molecular mechanisms of hepatic ischemia-reperfusion injury and preconditioning Am J Physiol Gastrointest Liver Physiol 2003 284 1 G15 G26 12488232 10.1152/ajpgi.00342.2002

[B33] Liu Z Xu Z Shen W Li Y Zhang J Ye X Effect of pharmacologic preconditioning with tetrandrine on subsequent ischemia/reperfusion injury in rat liver World J Surg 2004 28 6 620 624 15366756 10.1007/s00268-004-7172-3

[B34] Oreopoulos GD Wu H Szaszi K Fan J Marshall JC Khadaroo RG He R Kapus A Rotstein OD Hypertonic preconditioning prevents hepatocellular injury following ischemia/reperfusion in mice: a role for interleukin 10 Hepatology 2004 40 1 211 220 10.1002/hep.20281 15239105

[B35] Takahashi Y Tamaki T Tanaka M Konoeda Y Kawamura A Katori M Kakita A Efficacy of heat-shock proteins induced by severe fasting to protect rat livers preserved for 72 hours from cold ischemia/reperfusion injury Transplant Proc 1998 30 7 3700 3702 10.1016/S0041-1345(98)01201-9 9838624

[B36] Vajdova K Heinrich S Tian Y Graf R Clavien PA Ischemic preconditioning and intermittent clamping improve murine hepatic microcirculation and Kupffer cell function after ischemic injury Liver Transpl 2004 10 4 520 528 10.1002/lt.20126 15048795

[B37] Latchman DS Heat shock proteins and cardiac protection Cardiovasc Res 2001 51 4 637 646 10.1016/S0008-6363(01)00354-6 11530097

[B38] Parsell DA Lindquist S The function of heat-shock proteins in stress tolerance: degradation and reactivation of damaged proteins Annu Rev Genet 1993 27 437 496 10.1146/annurev.ge.27.120193.002253 8122909

[B39] Russell WJ Jackson RM MnSOD protein content changes in hypoxic/hypoperfused lung tissue Am J Respir Cell Mol Biol 1993 9 6 610 616 10.1165/ajrcmb/9.6.610 8257593

[B40] Knowlton AA Kapadia S Torre-Amione G Durand JB Bies R Young J Mann DL Differential expression of heat shock proteins in normal and failing human hearts J Mol Cell Cardiol 1998 30 4 811 818 10.1006/jmcc.1998.0646 9602430

[B41] Weisiger RA Fridovich I Superoxide dismutase, organelle specificity J Biol Chem 1973 248 10 3582 3592 4702877

[B42] Javadpour M Kelly CJ Chen G Bouchier-Hayes DJ Herbimycin-A attenuates ischaemia-reperfusion induced pulmonary neutrophil infiltration Eur J Vasc Endovasc Surg 1998 16 5 377 382 10.1016/S1078-5884(98)80003-8 9854547

[B43] Javadpour M Kelly CJ Chen G Stokes K Leahy A Bouchier-Hayes DJ Thermotolerance induces heat shock protein 72 expression and protects against ischaemia-reperfusion-induced lung injury Br J Surg 1998 85 7 943 946 10.1046/j.1365-2168.1998.00722.x 9692569

[B44] Chen G Kelly C Stokes K Wang JH Leahy A Bouchier-Hayes D Induction of heat shock protein 72 kDa expression is associated with attenuation of ischaemia-reperfusion induced microvascular injury J Surg Res 1997 69 2 435 439 10.1006/jsre.1997.5059 9224420

[B45] Stojadinovic A Kiang J Smallridge R Galloway R Shea-Donohue T Induction of heat-shock protein 72 protects against ischemia/reperfusion in rat small intestine Gastroenterology 1995 109 2 505 515 10.1016/0016-5085(95)90339-9 7615200

[B46] Weiss SJ Ward PA Immune complex induced generation of oxygen metabolites by human neutrophils Journal of immunology 1982 129 1 309 313 6282966

[B47] Heinzelmann M Mercer-Jones MA Passmore JC Neutrophils and renal failure Am J Kidney Dis 1999 34 2 384 399 10.1016/S0272-6386(99)70375-6 10430993

[B48] Zimmerman BJ Grisham MB Granger DN Role of oxidants in ischemia/reperfusion-induced granulocyte infiltration Am J Physiol 1990 258 2 Pt 1 G185 G190 2154938 10.1152/ajpgi.1990.258.2.G185

